# Who remembers the Beatles? The collective memory for popular music

**DOI:** 10.1371/journal.pone.0210066

**Published:** 2019-02-06

**Authors:** Stephen Spivack, Sara Jordan Philibotte, Nathaniel Hugo Spilka, Ian Joseph Passman, Pascal Wallisch

**Affiliations:** 1 Department of Psychology, New York University, New York, New York, United States of America; 2 Center for Neural Science, New York University, New York, New York, United States of America; University of L'Aquila, ITALY

## Abstract

How well do we remember popular music? To investigate how hit songs are recognized over time, we randomly selected number-one *Billboard* singles from the last 76 years and presented them to a large sample of mostly millennial participants. In response to hearing each song, participants were prompted to indicate whether they recognized it. Plotting the recognition proportion for each song as a function of the year during which it reached peak popularity resulted in three distinct phases in collective memory. The first phase is characterized by a steep linear drop-off in recognition for the music from this millennium; the second phase consists of a stable plateau during the 1960s to the 1990s; and the third phase, a further but more gradual drop-off during the 1940s and 1950s. More than half of recognition variability can be accounted for by self-selected exposure to each song as measured by its play count on Spotify. We conclude that collective memory for popular music is different from that of other historical phenomena.

## Introduction

Since the pioneering work of Ebbinghaus in 1885 [1], the study of individual memory has been a mainstay of cognitive psychology [2, 3]—even during the darkest days of behaviorism [4]. In contrast, collective memory—what groups of people or entire cultures know—has received comparatively little scientific attention [5, 6]. Yet, as exemplified by speculations about archetypes [7] and the collective consciousness [8], this question is of longstanding popular interest. An understanding of how cultures remember their past is of critical importance if we are to learn from history [9]. However, it is largely unknown how much history people remember.

Cognitive psychologists have begun to investigate this question in studies that probe collective memory for political leaders both in the United States [10] and in China [11]. Using a free recall paradigm, the results of these studies suggest the existence of a serial position effect [12] in collective memory, in which the recency portion shows a linear decline. We are not aware of existing literature that has investigated collective memory for other kinds of historical phenomena, such as popular music. Importantly, exposure to music is typically driven by personal interest; in other words, it is self-selective. Conversely, exposure to historical leaders is usually involuntary and often within academic settings, which could affect remembering over time [13, 14]. Another key distinction is that of set size, or how many items participants are expected to remember. For historical leaders, this number is finite and relatively small; in the United States there have only been 45 presidents to date. In contrast, for popular music this space is vast and essentially infinite; even the number of top singles on the *Billboard* popular music charts alone is well in excess of one-thousand since 1940 [15], which we think could rule out the possibility of a primacy effect in collective memory.

Given these considerations, we hypothesized that there might be a “cultural horizon” beyond which once popular music is effectively forgotten. Should this horizon exist, we aimed to characterize the drop-off as either linear [16] or exponential [1]. Finally, we were curious to know whether there are certain “evergreen” songs that are remembered regardless of how long ago they were first popular, akin to flashbulb memories [17]. In this paper, we aimed to empirically address each of these questions.

## Method

### Materials

We operationalized “popular music” as that which reached the number-one spot on the *Billboard* Top 100 between the years 1940 and 1957 and on the *Billboard* Hot 100 from 1958 to 2015 (we refer to both together as the *Billboard* henceforth). The *Billboard* is a record chart for the top singles in the United States and is the industry standard by which the popularity of contemporary music is measured. Rankings are published each week and are currently based on three components: record sales, radio airtime and online streaming [18]. We randomly selected two of the top songs from each year—for a total of 152 songs (see S1 Appendix for a list of the songs we used)—and presented them to participants via Audio-Technica ATH-M20x Professional Monitor Headphones using custom-built MATLAB (2016b) software (Mathworks, Natick, MA). Note that this sampling method is not weighed by time on chart and could be considered biased if this mattered, that is, if the time on chart was non-stationary over time. For instance, songs from the 1960s through 1980s stayed in the top spot, on average, for a much shorter time than in the 2000s. However, there is no empirical correlation between recognition proportion and length of time at the top of the charts in our sample, *r*(138) = -0.01, *p* = 0.94, so this is a fair sampling method. As a proxy for self-selected exposure to music, we recorded the play count for each song as it appeared on Spotify—a streaming service with the world’s largest collection of digital music, with over 140 million users [19]—as of October 8^th^, 2017. We also recorded the number of covers and samples for each song as they appeared on whosampled.com—a comprehensive and publicly accessible database—as of June 18^th^, 2018.

### Participants and task

To satisfy statistical power needs in both psychology and neuroscience [20], we used a sufficiently large sample to address our research questions. Each participant (*n* = 643) was presented with a random selection of seven out of the 152 songs and asked to listen to the selection and report whether they recognized it. Participants were also presented with 5-, 10- and 15-second excerpts deemed to be representative by a consensus panel of seven practicing musicians and professors of music theory and composition and often containing a highly recognizable “lick”—a unique and often repeated pattern of notes played by a single instrument—of each song. To control for the possibility of exposure effects on recognition, all songs and clips were presented in random order. Participants were recruited from the New York University student population for course credit as well as the greater New York metropolitan area, who were compensated for their time at $10 an hour. There were no meaningful differences in any of the measures reported in this manuscript between these two populations. Our sample consisted mostly of young participants, with a mean age of 21.3 years, a median age of 20 years and a standard deviation of 5.09 years. The majority (88%) of this sample was between the ages of 18 and 25, which we considered to be “millennials”. All experimental procedures were approved by the New York University Institutional Review Board, the University Committee on Activities Involving Human Subjects (UCAIHS). All participants provided their written informed consent prior to participating in the study.

### Data analysis

630 participants (98% of the total sample) completed the entire study and were used in this analysis. As much of our data is nonlinear, we used Spearman’s rho [21] to quantify the strength of the relationship between any two variables, such as song recognition and play count. To confirm the validity of our song recognition assay, we used a pairwise t-test to determine whether there are statistically significant differences between recognition for songs compared to clips of 5-, 10- and 15-second durations. In some cases, a pairwise t-test was not appropriate. When comparing phases, the unit of analysis is a song. However, the samples are not independent and the *n* is not matched between phases, as they contain an unequal number of songs. Instead, to determine whether there are statistically significant differences between the parameters—such as recognition proportion—for each phase, we remained conservative and used a Mann-Whitney U test [22]. Finally, we performed a normalized multiple linear regression to account for recognition as a function of Spotify play counts, number of covers and number of samples per song. To guard against false positives and to compensate for multiple comparisons, we adopt a significance level of 0.01. This is adequate, as our study is sufficiently powered. We would like to emphasize that we used participants of all ages in the analysis that is presented here. When restricting the same analysis to millennials (aged 18–25, *n* = 564), we found that there is no meaningful difference in our results when compared to our entire participant pool—only minor numerical differences. This is probably due to the fact that our full sample overwhelmingly consisted of participants in that age range. Spotify’s application programming interface did not contain play count data for two of the 152 songs, so we did not include them in the analysis of Spotify playcounts. Similarly, whosampled.com did not contain data for 14 of these songs, so we did not include those in the analysis of covers and samples.

## Results

**Fig 1:**
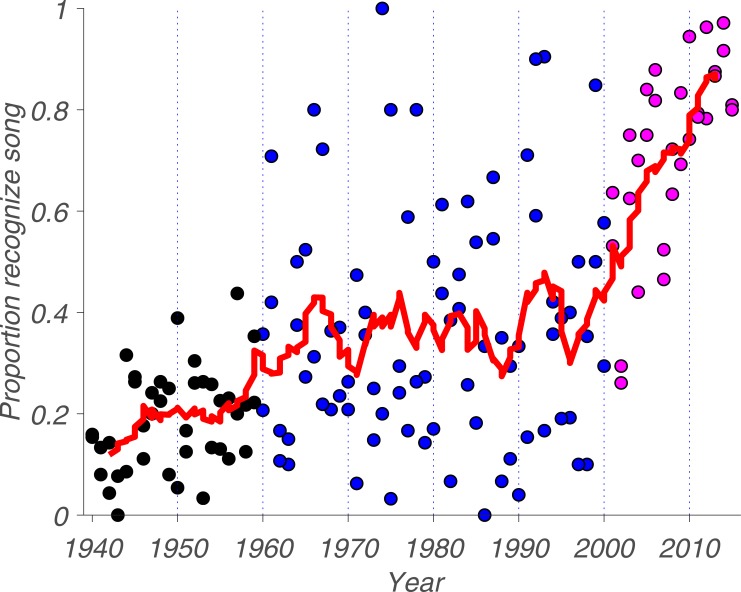
Recognition of songs over time. Dots represent the proportion of participants who reported to recognize a given song from a given year. The red curve represents the convolved average proportion for a given year, integrating over 5 years. Magenta dots represent songs from Phase 1 (2001 to 2015), blue dots represent songs from Phase 2 (1960 to 2000) and black dots represent songs from Phase 3 (1940 to 1959). Dashed vertical lines are decade markers.

The main question of this paper is whether there is a cultural horizon for the collective memory for popular music. If such a horizon were to exist, we also wanted to know at what point in time it occurs and whether it is approached linearly or exponentially. To answer these questions, we calculated the proportion of participants who reported to recognize each song and plotted this proportion as a function of the year it reached the number-one spot on the *Billboard* (Fig 1).

As assessed by Spearman’s rho, the proportion of participants who reported to recognize a given song increases with how recently that song occupied the number-one spot on the *Billboard* popular music charts, *r*_*s*_(150) = 0.62, *p* = 1.70e-17. This is not surprising and could be considered a manipulation check. As can be seen in Fig 1, this trend is far from linear. Whereas the average recognition proportion across all songs and all years is 0.39, we observed three distinct phases in collective memory. Phase 1: recognition was high but decreased steeply and linearly from 2015 until the turn of the millennium. Phase 2: there was a stable plateau at an average recognition proportion of 0.37 from the late 1990s to the early 1960s. Phase 3: starting in the late 1950s, recognition was quite low and slowly decreased toward oblivion.

**Fig 2:**
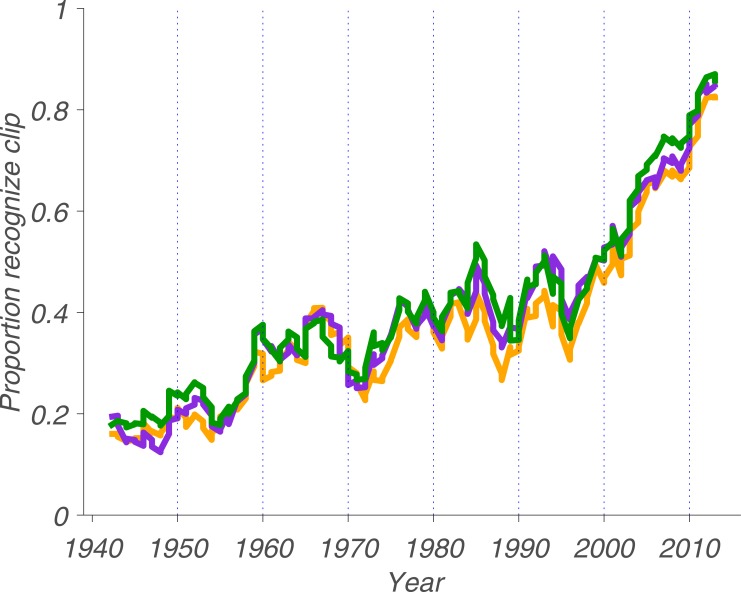
Recognition of clips over time. Orange curve: 5 second clips, purple curve: 10 second clips, green curve: 15 second clips. All three curves represent the convolved average proportion for a given year, integrating over 5 years. Dashed vertical lines are decade markers.

As we asked our participants to indicate whether they recognized each song in response to hearing it, we probed the *feeling* of recognition at the time they heard it—not whether they could accurately select the title from a list of related titles. This raises the question of whether our assay is a valid means of capturing recognition memory. To confirm the validity of this method, we replicated the above finding using short excerpts (5-, 10- or 15-second clips) that we deemed to be representative of each song (Fig 2).

As assessed by Spearman’s rho, recognition for clips is remarkably similar to that of songs, regardless of clip duration, *r*_*s*_(150) = 0.97, *p* = 1.12e-89. Indeed, when we used a pairwise t-test we found that none of the clip durations yielded recognition rates that are statistically distinguishable from that of the songs, *t*(151) = 1.29, *p* = 0.20, d = 0.05; *t*(151) = 1.49, *p* = 0.14, d = 0.05; and *t*(151) = 2.36, *p* = 0.02, d = 0.10, for 5-, 10- and 15-seconds, respectively. In absolute terms, the mean recognition for 5-second clips is 0.38, for 10-second clips is 0.40 and for 15-second clips is 0.42. Using a pairwise t-test to compare different clip durations against one another we found that the difference between the recognition proportions for 5-second clips and 10-second clips is statistically significant, *t*(151) = 3.18, *p* = 0.0018, d = 0.14; the difference between 5-second and 15-second clips is also statistically significant, *t*(151) = 4.42, *p* = 1.88e-05, d = 0.10; but the difference between 10-second and 15-second clips is not, *t*(151) = 1.26, *p* = 0.21, d = 0.04. This is visually evident in Fig 2, as the orange trace tends to be below the purple trace, which in turn tends to be below the green trace—but the effect sizes are small. Therefore, it seems that five seconds is enough time for participants to accurately report whether they recognized a given song from a clip. This is consistent with the finding that, in response to hearing 400-millisecond “thin slices” of music, people are able to identify both the song title and artist [23]. Moreover, it has also been shown that people are able to reliably categorize music genres in as little as 250 milliseconds of hearing a song [24]. Thus, as there is no difference in recognition between clips and songs, we conclude that we used a valid metric of recognition memory—participants responded far from randomly.

**Fig 3:**
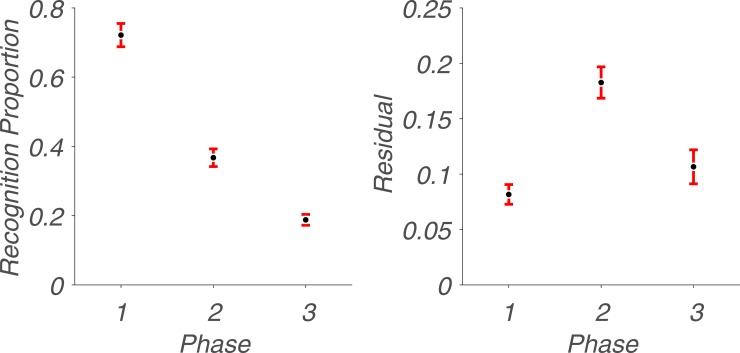
Quantifying average song recognition proportions and their variability over the three phases. Left panel: Black dots represent the mean recognition rate for a given phase. Red bars represent the standard error of the mean. Right panel: Black dots represent the average residual—the distance between individual songs to the corresponding point on the convolved average (red curve) from Fig 1, for a given phase. Red bars represent the standard error of the mean.

For the next part of our analysis (Fig 3), we quantified the mean song recognition proportion and mean variability—defined here as the average residual from the convolved mean—for each of the three phases we previously identified in Fig 1.

The mean recognition proportions for each of the three phases are 0.72, 0.37 and 0.19, respectively. To confirm that the difference between each phase is statistically significant, we performed a Mann-Whitney U test to compare Phases 1 and 2, U = 2140, *p* = 2.28e-09; Phases 2 and 3, U = 14.5, *p* = 3.83e-12; and phases 1 and 3, U = 840.5, *p* = 1.31e-05. Indeed, the proportions for each of these three phases have a statistically significant difference from one another, with a general downward trend in recognition across time.

For the mean residual of each phase, we observed a different pattern: during Phase 1—and to a lesser extent Phase 3—the recognition proportion of individual songs closely follows the mean for a given year. In contrast, inter-song variability is high during Phase 2, where the mean does not closely represent recognition rates of individual songs. Some songs in this phase are recognized extremely well, such as “When A Man Loves A Woman” by Percy Sledge (1966) whereas others like “Knock Three Times” by Dawn (1971) are all but forgotten. The mean residual for each phase is 0.08, 0.18 and 0.11, respectively. When we performed a Mann-Whitney U test to determine whether this observation is supported by the data, we found that Phases 1 and 2 have a statistically significant difference, U = 696, *p* = 0.008; that Phases 2 and 3 have a statistically significant difference, U = 707, *p* = 1.49e-05; but that Phrases 1 and 3 do not, U = 527, *p* = 0.30. Thus, recognition variability is highest during Phase 2. This raises the question of what drives the variability in recognition, both between phases and between songs within a given phase. One possible explanation for the former is simply exposure: people are more likely to recognize songs to which they have been exposed more often [25].

**Fig 4:**
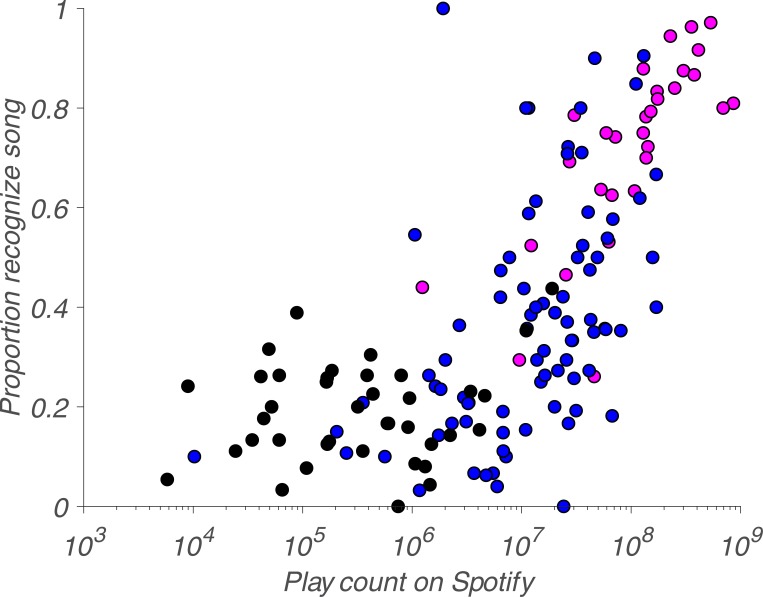
Song recognition versus play count. Magenta dots represent songs from Phase 1 (2001 to 2015), blue dots represent songs from Phase 2 (1960 to 2000) and black dots represent songs from Phase 3 (1940 to 1959).

To address the question of whether exposure can account for a sufficiently large proportion of recognition variability, we used play counts on a digital streaming service—in this case, Spotify—as a proxy for self-selected exposure to music and plotted them against recognition proportion (Fig 4).

As assessed by Spearman’s rho, there is a considerable correlation between the likelihood of recognizing a given song and its corresponding play count on Spotify, *r*_*s*_(148) = 0.73, *p* = 6.09e-27. Thus, a substantial amount of the variability in recognition proportion can be accounted for by this metric of self-selected exposure to each song. This is remarkable given that Spotify was launched in 2008, well after the majority (89%) of this music was released, suggesting that the relatively young cohort of participants in our study consumes music primarily through digital streaming. Of course, Spotify is only one proxy of exposure; there are others, such as the number of covers or samples, which presumably are also considerable sources of exposure to the original song. When assessing this possibility with a regression model we note that most of the variability in recognition proportion in our sample is captured by Spotify play counts, but the number of samples and number of covers also explains smaller but significant proportions of variance in recognition, R^2^ = 0.41, F(2,134) = 30.93, p <0.01. The respective normalized beta-coefficients are 0.48, 0.24 and 0.14, for Spotify play counts, number of samples and numbers of covers. However, note that one ought to be cautious when interpreting these numbers at face value, as it presumes that songs were covered and sampled at random, which is presumably not the case. There is a high likelihood why some songs are covered more often than others, including the sheer possibility that they are covered more in the future because they have been covered more in the past. In other words, being covered is highly self-selective and until further research uncovers the reasons for why some songs are covered and others are not, it is hard to interpret what this means.

Given the results of research on collective memory for other historical phenomena, we would not have expected the stable plateau—characterized by high within-phase recognition variability—that we found. One possibility is that this phase results from our participants listening to the music of their parents, when growing up in their household, as suggested by studies on the autobiographical memory of music [26–28]. Another possibility is that the music from the 1960s onwards truly was different from earlier music, with music from the 1960s to the 1990s representing a particularly special time in music. We know from the history of music that it did change dramatically from the 1960s onward—the common practice period transitioned into rock music and then electronically-generated music (Ted Coons, personal communication, 01/23/2018). Moreover, the lyrics of popular music also changed. Starting in the 1960s, we saw political music, whereas prior to that time, love songs dominated, as shown by a content analysis [29]. These possibilities are not mutually exclusive, but we were curious to know if we could find further support for the notion that the 1960s to 1990s were a unique time in terms of popular music.

**Fig 5:**
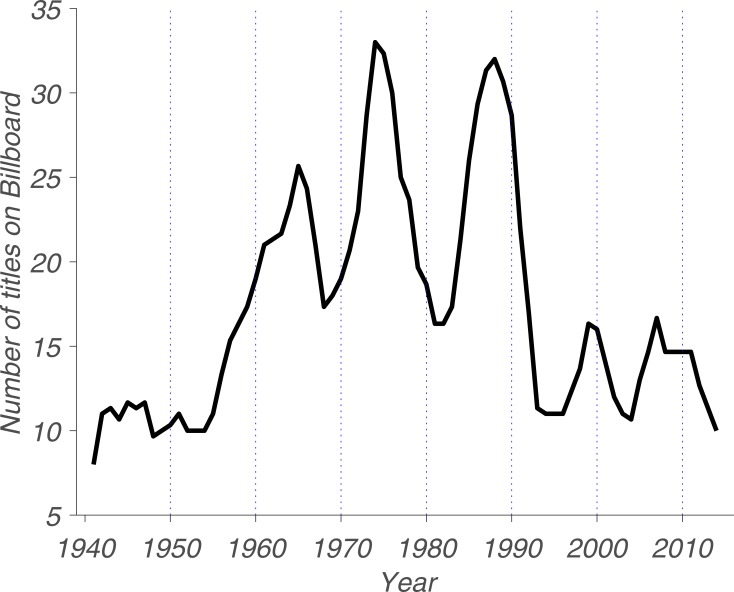
Diversity of music over time. Note that the data was smoothed by a 3-year moving average. A value of just above 10 in the early 1940s means that an average song was at the top of the charts for over 4 weeks in a row. The peak levels in the mid-1970s mean that the average song was only on top of the charts for little more than a week during that period.

To address this issue, we looked at the diversity of song titles over time. To quantify this trend, we plotted the number of unique titles that occupied the number-one spot as a function of the year during which they were most popular. In years during which this number was low, few titles managed to hog the top spot; in years during which there was fierce competition between equally popular but different songs, this number was high, as it was more difficult for any given song to stay on top for long (Fig 5).

We observed three distinct phases similar to those seen in Fig 1. Phase 1: low diversity from 2015 to the 1990s. Phase 2: high diversity from the 1960s to the 1990s. Phase 3: low diversity—again—from the 1940s to the end of the 1950s. Diversity here means the number of unique singles that occupied the number-one spot during a given year. To quantify the diversity in titles over time, we first binned the data according to the phases we identified in Fig 1 and then computed the mean and standard error of the mean for each. The mean for each of the three phases is 6.73, 11.32 and 6.14, respectively. Next, we performed a Mann-Whitney U test to confirm that there is, once again, a statistically significant difference between Phases 1 and 2, U = 83, *p* = 3.32e-05; and Phases 2 and 3, U = 71, *p* = 1.88e-07; but not Phases 1 and 3, U = 192, *p* = 0.16. This pattern is undeniable, but we cannot speculate the underlying reasons. Anything we could propose, such as changes in listening habits and technology or the rise of DJ culture and dance music, would be purely speculative. However, there seem to be distinct diversity peaks in almost every decade, which could lead to the suggestion of “boom and bust” cycles in music, giving each decade a unique sound when the old sound has “played out”.

## Discussion

We identified three distinct phases in the collective memory for popular music. The first phase is characterized by generally high recognition with a steep linear drop-off and low inter-song variability. For the second phase, we observed moderately high recognition with high inter-song variability—some songs are well-remembered whereas others are not. In the third and final phase, recognition drops again to rather low levels as time passes, and inter-song variability is relatively low again. We also found that a sizeable proportion—more than half—of this recognition variability can be accounted for by self-selected exposure to each song as measured by its overall play count on Spotify.

We interpret these findings to mean that collective memory for popular music—and perhaps for cultural artifacts in general—is different than that for political leaders, be they in the United States [10] or China [11]. There are two important differences. First, the drop-off we observed is only linear for a short period, until it hits the recognition plateau from the 1960s to the 1990s. Second, there seems to be no primacy effect for popular music. There are several possible explanations for this. For instance, the primacy effect for political leaders presumably stems from the fact that the founders of a political dynasty are frequently repeated in public discourse. Conversely, people demonstrably do not choose to listen to songs from the early years of the *Billboard*, if Spotify play counts are a valid metric of exposure. In fact, play counts for this period are remarkably low (see Fig 4). The issue of play counts highlights another difference between existing work on collective memory for political leaders and our work here. Presumably, people are exposed to political leaders in history classes and coursework, much of which is part of mandatory schooling. In contrast, much—if not most—of music listening is both voluntary and self-selective. There are other differences as well. The number of historical leaders is countably finite—in terms of United States presidents, there have been only 45 to date. However, the universe of existing music is vast and—from the perspective of an individual—basically boundless. Given this consideration, it is surprising that our recognition rates are as high as they are. As there are strong recency effects for both Chinese and American leaders—but not for popular music—the actionable advice would be that if one wants to remain famous in politics, they must found a political movement. Conversely, if one wants to remain famous for their art, they have to keep producing new releases, or release them during a special period that increases the chances of being remembered. Few things will stand the test of time, in particular if they are not repeated. Finally, our work involved reports of recognition in response to a specific stimulus whereas most existing work on collective memory used free recall. One paper that did use recognition methods—again on United States political leaders—suggests a proneness to false-positives due to contextual familiarity [30], as has been shown previously for other material [31].

In addition, these results put the notion of a hard cultural horizon in question. We do not find a period during which recognition drops to zero. Even our millennial participants—which formed the bulk of our participant pool—were not completely unaware of the music that was popular in the 1940s. We posit several plausible explanations for this. For instance, it is possible that not enough time has passed since the first titles of our stimuli set. Perhaps a future study might be able to identify when recognition hits zero (the cultural horizon), if there is a long enough history of *Billboard* music. However, it is also possible that involuntary exposure through film, television and radio or accidental exposure through music discovery services such as Pandora allows people to discover music that might be very old indeed. Finally, people might also intentionally seek out the music of previous generations. In principle—as much, if not all music is recorded and digitally available forever—this could prevent a cultural horizon indefinitely. We are unable to distinguish these possibilities in the present study.

This study has other limitations as well. For instance, we used Spotify play counts, numbers of covers and numbers of samples as a proxy for total music exposure. We do not have data on how often individuals listen to music in analog format, on other web-based platforms, on the radio, or are exposed to music in everyday life in other forms such as movie and television soundtracks, or simply as background music. Put differently, all of the observed recognition effects might be entirely driven by exposure, but our measure of exposure is noisy if not biased, lowering the proportion of variance in recognition rates for which we cannot account. In addition, it is possible that newer music has greater play counts on Spotify for several reasons, none of which are mutually exclusive, and none of which we can distinguish. For instance, older music might be more readily available in analog formats such as cassette tapes or vinyl records, which could lower the digital play count of such music. Conversely, it is also possible that the average Spotify user—who is between the age of 13 and 29 [18]—preferentially listens to the music of their generation. In this cohort, that corresponds to music from the 2000s and 2010s. However, this line of reasoning is not central to our argument that distinct phases exist. That we can account for as much of the variance in recognition as we do, simply with Spotify play counts, does suggest that this is a reasonable proxy for exposure in general, and that voluntary exposure through digital streaming is a main driver of recognition. This does not preclude that there are other factors that influence recognition as well. Music is a complex phenomenon and songs are not unaffected by the socio-cultural context in which they are released, and this might in turn impact how they are remembered. A related limitation is that most of our participants were young adults. Presumably, if we had multiple age cohorts, the linear recency peaks would average out, perhaps yielding an overall flat recognition curve. In that sense, it was good that our sample overrepresented a single generation. We predict that future studies with multiple age cohorts will exemplify distinct and shifted recency peaks that are unique to a given age cohort. A final limitation worth noting is that we did not test recognition strictly speaking—but rather the feeling of recognition—as we asked participants whether they recognized what they heard, not whether they could select the title of the song from a list of related titles. Whether this report is actually accurate remains unknown. However, we would like to emphasize that our findings have face validity. It would be surprising if our cohort of millennials reported higher recognition rates for music before their time. Yet, consistent with previous research on memory for popular music [32] and historical events [33] that date prior to one’s lifetime, we did not observe such recognition reports. Moreover, the strong correlation with exposure and the concordance between clip and song recognition patterns would be very surprising if participants responded randomly. Thus, we conclude that this measure of recognition is reasonably valid. To get an estimate of the false recognition rate, it would be advisable to include a piece of unpublished music in the stimulus set as a lure. Finally, note that we use the term “collective memory” sensu Roediger, i.e. in terms of what large groups of people remember and forget. In other words, what a group remembers is conceptually the average recall of the individuals that make up the group and in terms of music can be largely explained in terms of the exposure of individuals (as exemplified by Spotify playcounts). There is an important other way in which the term is used. For some social scientists, collective memories transcend individuals, whereas others recognize that even in that case, individuals are the carriers of these shared memories [34]

Taken together, the most compelling interpretation supported by our data is the notion that the period from the 1960s to the 1990s was a special time in popular music history. During this period, we observe an extended plateau characterized by relatively high recognition that is surprising given prior research on collective memory. However, to determine whether the music of the 1960s to 1990s was truly special will require a follow-up study in another 30 years or so. Our prediction is that in another generation, people will still preferentially listen to current music, but the linear drop off phase will be extended as the 1960s to 1990s plateau shifts back in time. The recognition curve might even have multiple peaks by then, as the plateau we identified in the current study will look much like a primacy effect, if the music of the 1960s to the 1990s appeals to the children of millennials. Conversely, if the autobiographical memory explanation is true [28,35,36], the general pattern should look much like it does now, only with an extended Phase 3, that perhaps could even reach close to zero recognition.

## Conclusion

In this paper, we investigated the collective memory for popular music. Studies on collective memory for other historical phenomena suggest the existence of a serial position curve, in which the recency portion shows a linear decline. However, due to the inherent self-selective nature of music listening behavior, we reasoned that memory for popular music might be different. Specifically, we hypothesized the existence of a cultural horizon beyond which memory for once popular music is effectively forgotten. Although our data support the existence of the recency portion of the serial position curve, we see neither a primacy effect nor a cultural horizon for the earliest music. Instead, our large sample of mostly millennial participants seemed to remember at least some of the hit songs from the 1940s and 1950s. Thus, we conclude that collective memory for popular music is different from that of other historical phenomena.

## Supporting information

S1 Appendix List of *Billboard* Songs. For each song we used in our study, we included the year, song title, artist, mean recognition proportion and play count on Spotify (in millions).
(PDF)
