# Supplementary material for: Who remembers the Beatles? The collective memory for popular music
Source: PLoS One. 2019 Feb 6;14(2):e0210066. doi: 10.1371/journal.pone.0210066 (PMC6364888; doi:10.1371/journal.pone.0210066)
Supplement: S1 Appendix — For each song we used in our study, we included the year, song title, artist, mean recognition proportion and play count on Spotify (in millions). (PDF) [file pone.0210066.s001.pdf]

S1 Appendix

Year	Song	Artist	Recognition	Play count (M)
2015	What Do You Mean?	Justin Bieber	0.81	859.455
2015	See You Again	Wiz Khalifa ft. Charlie Puth	0.80	693.251
2014	All About That Bass	Meghan Trainor	0.92	411.734
2014	Happy	Pharrell Williams	0.97	533.017
2013	Wrecking Ball	Miley Cyrus	0.88	300.566
2013	Locked Out of Heaven	Bruno Mars	0.87	377.360
2012	Diamonds	Rihanna	0.96	355.862
2012	Stronger (What Doesn't Kill You)	Kelly Clarkson	0.78	136.752
2011	Black and Yellow	Wiz Khalifa	0.79	151.211
2011	Hold It Against Me	Britney Spears	0.79	30.247
2010	Not Afraid	Eminem	0.94	228.394
2010	OMG	Usher ft. Will.I.Am.	0.74	71.893
2009	Fireflies	Owl City	0.83	172.616
2009	"3"	Britney Spears	0.69	27.400
2008	Whatever You Like	T.I.	0.63	107.525
2008	Bleeding Love	Leona Lewis	0.72	142.313
2007	Give It To Me	Timbaland	0.47	25.404
2007	This Is Why I'm Hot	Mims	0.52	12.224
2006	Irreplaceable	Beyonce	0.88	129.371
2006	SexyBack	Justin Timberlake	0.82	174.620
2005	Gold Digger	Kanye West ft. Jamie Foxx	0.84	250.227
2005	Hollaback Girl	Gwen Stefani	0.75	129.048
2004	Drop It Like It's Hot	Snoop Dogg ft. Pharrell	0.70	137.306
2004	I Believe	Fantasia	0.44	1.239
2003	Baby Boy	Beyonce ft. Sean Paul	0.75	59.249
2003	Get Busy	Sean Paul	0.63	66.823
2002	Foolish	Ashanti	0.26	45.958
2002	Ain't It Funny	Ja Rule & Jennifer Lopez	0.29	9.535
2001	Family Affair	Mary J. Blige	0.53	62.546
2001	Bootylicious	Destiny's Child	0.64	53.028
2000	Come On Over Baby (All I Want Is You)	Christina Aguilera	0.29	13.801
2000	Smooth	Santana ft. Rob Thomas	0.58	68.081
1999	Bills, Bills, Bills	Destiny's Child	0.50	32.467
1999	...Baby One More Time	Britney Spears	0.85	110.882
1998	Lately	Divine	0.10	0.563
1998	Truly Madly Deeply	Savage Garden	0.35	80.557
1997	4 Seasons of Loneliness	Boyz II Men	0.10	7.211
1997	Hypnotize	Notorious B.I.G.	0.50	157.181
1996	No Diggity	Blackstreet ft. Dr. Dre	0.40	169.896
1996	One Sweet Day	Mariah Carey & Boyz II Men	0.19	31.435
1995	Exhale (Shoop Shoop)	Whitney Houston	0.19	6.748
1995	Have You Ever Really Loved A Woman?	Bryan Adams	0.39	20.164
1994	I Swear	All-4-One	0.42	23.837
1994	Bump N' Grind	R. Kelly	0.36	57.611
1993	Informer	Snow	0.17	26.780
1993	I Will Always Love You	Whitney Houston	0.90	130.382
1992	Baby Got Back	Sir Mix-A-Lot	0.90	46.558
1992	Jump	Kriss Kross	0.59	40.487
1991	Joyride	Roxette	0.15	10.877
1991	Gonna Make You Sweat (Everybody Dance Now)	C&C Music Factory	0.71	35.426
1990	Praying For Time	George Michael	0.04	5.976
1990	How Am I Supposed To Live Without You?	Michael Bolton	0.33	29.067
1989	If You Don't Know Me By Now	Simply Red	0.29	25.694
1989	Lost In Your Eyes	Debbie Gibson	0.11	6.797
1988	Every Rose Has Its Thorn	Poison	0.35	45.721
1988	Roll With It	Steve Winwood	0.07	3.677
1987	With Or Without You	U2	0.67	169.532
1987	Lean On Me	Club Nouveau	0.55	1.059
1986	The Way It Is	Bruce Hornsby and the Range	0.00	24.203
1986	Holding Back The Years	Simply Red	0.33	28.593

1985	Money For Nothing	Dire Straits	0.18	67.141
1985	Heaven	Bryan Adams	0.54	60.783
1984	Caribbean Queen	Billy Ocean	0.26	30.095
1984	Footloose	Kenny Loggins	0.62	120.084
1983	Maniac	Michael Sembello	0.48	42.039
1983	Flashdance... What A Feeling	Irene Cara	0.41	15.736
1982	Truly	Lionel Richie	0.07	5.492
1982	Abracadabra	The Steve Miller Band	0.38	12.166
1981	Kiss On My List	Daryl Hall & John Oates	0.44	10.478
1981	The Tide Is High	Blondie	0.61	13.495
1980	Magic	Olivia Newton-John	0.17	3.112
1980	Rock With You	Michael Jackson	0.50	49.218
1979	Sad Eyes	Robert John	0.14	1.745
1979	My Sharona	The Knack	0.27	41.390
1978	Shadow Dancing	Andy Gibb	0.26	1.422
1978	Baby Come Back	Player	0.80	11.581
1977	You Light Up My Life	Debby Boone	0.17	2.310
1977	Gonna Fly Now	Bill Conti	0.59	11.599
1976	Love Machine	The Miracles	0.29	2.010
1976	Saturday Night	Bay City Rollers	0.24	1.631
1975	I'm Sorry	John Denver	0.03	1.168
1975	Lady Marmalade	Labelle	0.80	10.961
1974	Can't Get Enough Of Your Love, Babe	Barry White	0.20	19.997
1974	The Loco-Motion	Grand Funk	1.00	1.918
1973	The Most Beautiful Girl	Charlie Rich	0.15	6.771
1973	Top Of The World	Carpenters	0.25	15.001
1972	Ben	Michael Jackson	0.40	13.490
1972	Heart Of Gold	Neil Young	0.36	58.684
1971	Gypsies, Tramps, & Thieves	Cher	0.47	6.448
1971	Knock Three Times	Dawn	0.06	4.736
1970	The Long and Winding Road	The Beatles	0.26	16.313
1970	Everything is Beautiful	Ray Stevens	0.21	0.355
1969	Wedding Bell Blues	The 5th Dimension	0.24	1.823
1969	Get Back	The Beatles w/ Billy Preston	0.37	26.074
1968	Love Child	Diana Ross & The Supremes	0.21	3.290
1968	Harper Valley P.T.A.	Jeannie C. Riley	0.36	2.699
1967	Hello Goodbye	The Beatles	0.72	26.566
1967	The Happening	The Supremes	0.22	2.960
1966	When A Man Loves A Woman	Percy Sledge	0.80	34.599
1966	Monday, Monday	The Mamas & The Papas	0.31	15.992
1965	Help!	The Beatles	0.52	36.127
1965	Eight Days A Week	The Beatles	0.27	21.438
1964	Leader Of The Pack	The Shangri-Las	0.50	7.741
1964	Love Me Do	The Beatles	0.38	42.738
1963	Easier Said Than Done	The Essex	0.10	0.010
1963	Go Away Little Girl	Steve Lawrence	0.15	0.205
1962	Soldier Boy	The Shirelles	0.17	0.593
1962	Don't Break The Heart That Loves You	Connie Francis	0.11	0.251
1961	The Lion Sleeps Tonight	The Tokens	0.71	26.213
1961	Blue Moon	The Marcels	0.42	6.401
1960	Are You Lonesome Tonight?	Elvis Presley w/ the Jordanaires	0.36	11.192
1960	I'm Sorry	Brenda Lee	0.21	3.242
1959	Sleepwalk	Santo & Johnny	0.22	4.605
1959	The Chipmunk Song	The Chipmunks w/ David Seville	0.35	11.012
1958	It's Only Make Believe	Conway Twitty	0.13	1.500
1958	It's All in the Game	Tommy Edwards	0.22	0.946
1957	All Shook Up	Elvis Presley	0.44	18.986
1957	Round and Round	Perry Como	0.20	0.319
1956	I Want You, I Need You...	Elvis Presley	0.23	3.416
1956	The Wayward Wind	Gogi Grant	0.11	0.024
1955	The Yellow Rose of Texas	Mitch Miller	0.23	0.439
1955	Cherry Pink (and Apple...)	Perez Prado w/ his orchestra	0.13	0.175
1954	I Need You Now	Eddie Fisher	0.13	0.061
1954	Oh! My Pa-Pa	Eddie Fisher	0.26	0.167

1953	I'm Walking Behind You	Eddie Fisher	0.26	0.061
1953	Till I Waltz Again	Teresa Brewer	0.03	0.065
1952	Why Don't You Believe Me?	Boni James	0.26	0.041
1952	Wheel of Fortune	Kay Starr	0.30	0.418
1951	Cold, Cold Heart	Tony Bennett	0.13	0.168
1951	How High The Moon	Les Paul & Mary Ford	0.17	0.608
1950	The Thing	Phil Harris	0.39	0.088
1950	Rag Mop	Ames Brothers	0.05	0.006
1949	That Lucky Old Sun	Frankie Laine	0.25	0.165
1949	A Little Bird Told Me	Evelyn Knight	0.08	N/A
1948	Buttons and Bows	Dinah Shore	0.26	0.388
1948	Twelfth Street Rag	Pee Wee Hunt w/ his orchestra	0.23	N/A
1947	Ballerina	Vaughn Monroe w/ his orchestra	0.24	0.009
1947	Near You	Francis Craig w/ his orchestra	0.20	0.052
1946	The Gypsy	The Ink Spots	0.11	0.354
1946	I Can't Begin To Tell You	Bing Crosby & Carmen Cavallaro	0.18	0.044
1945	My Dreams Are Getting Better...	Les Brown w/ his orchestra	0.27	0.185
1945	Don't Fence Me In	Bing Crosby & The Andrews Sisters	0.26	0.793
1944	I'm Making Believe	The Ink Spots & Ella Fitzgerald	0.09	1.065
1944	Besame Mucho (Kiss Me Much)	Jimmy Dorsey w/ his orchestra	0.32	0.049
1943	I've Heard That Song Before	Harry James w/ his orchestra	0.00	0.744
1943	I Had The Craziest Dream	Harry James w/ his orchestra	0.08	0.108
1942	Jingle, Jangle, Jingle	Kay Kryser w/ his orchestra	0.04	1.454
1942	A String Of Pearls	Glenn Miller w/ his orchestra	0.14	2.223
1941	Chattanooga Choo Choo	Glenn Miller w/ his orchestra	0.08	1.314
1941	Daddy	Sammy Kaye w/ his orchestra	0.13	0.035
1940	Frenesi	Artie Shaw w/ his orchestra	0.16	0.917
1940	I'll Never Smile Again	Tommy Dorsey w/ his orchestra	0.15	4.120
